# Calcium Overload or Underload? The Effects of Doxorubicin on the Calcium Dynamics in Guinea Pig Hearts

**DOI:** 10.3390/biomedicines10092197

**Published:** 2022-09-06

**Authors:** Jingjing Wu, Linlin Gao, Hong Fan, Deming Liu, Mengxue Lin, Ming Zhu, Tian Deng, Yuanlong Song

**Affiliations:** 1 Department of Cardiology, Huazhong University of Science and Technology, Wuhan 430022, China; 2 Hubei Key Laboratory of Biological Targeted Therapy, Huazhong University of Science and Technology, Wuhan 430022, China; 3 Hubei Provincial Engineering Research Center of Immunological Diagnosis and Therapy for Cardiovascular Diseases, Union Hospital, Tongji Medical College, Huazhong University of Science and Technology, Wuhan 430022, China; 4 Department of Physiology, Tongji Medical College, Huazhong University of Science and Technology, Wuhan 430030, China; 5 Department of Emergency Medicine, Union Hospital, Tongji Medical College, Huazhong University of Science and Technology, Wuhan 430022, China; 6 National Engineering Laboratory for Next-Generation Internet Access System (NEL-NGIA), Huazhong University of Science and Technology, Wuhan 430022, China; 7 School of Electronic Information and Communications, Huazhong University of Science and Technology, Wuhan 430074, China

**Keywords:** doxorubicin toxicity, intracellular Ca^2+^, optical mapping

## Abstract

The severe doxorubicin (DOXO) side effect of cardiomyopathy limits it clinical application as an effective anticancer drug. Although Ca^2+^ overload was postulated as one of the mechanisms for this toxicity, its role was, however, disputable in terms of the contractile dysfunction. In this work, the dynamics of the intracellular Ca^2+^ signal were optically mapped in a Langendorff guinea pig heart. We found that DOXO treatment: (1) Delayed the activation of the Ca^2+^ signal. With the reference time set at the peak of the action potential (AP), the time lag between the peak of the Ca^2+^ signal and AP (Ca-AP-Lag) was significantly prolonged. (2) Slowed down the intracellular Ca^2+^ releasing and sequestering process. Both the maximum rising (MRV) and falling (MFV) velocity of the Ca^2+^ signal were decreased. (3) Shortened the duration of the Ca^2+^ signal in one cycle of Ca^2+^ oscillation. The duration of the Ca^2+^ signal at 50% amplitude (CaD50) was significantly shortened. These results suggested a reduced level of intracellular Ca^2+^ after DOXO treatment. Furthermore, we found that the effect of tachypacing was similar to that of DOXO, and, interestingly, DOXO exerted contradictory effects on the tachypaced hearts: it shortened the Ca-AP-Lag, accelerated the MRV and MFV, and prolonged the CaD50. We, therefore, concluded that DOXO had a different effect on intracellular Ca^2+^. It caused Ca^2+^ underload in hearts with sinus rhythm; this might relate to the contractile dysfunction in DOXO cardiomyopathy. It led to Ca^2+^ overload in the tachypaced hearts, which might contribute to the Ca^2+^-overload-related toxicity.

## 1. Introduction

Doxorubicin (DOXO) is a widely used chemotherapy medication for the treatment of cancer. Its toxicity on the heart has been drawing research attention for decades [1,2,3]. Dilated cardiomyopathy is the most dangerous side effect, which can lead to congestive heart failure. This contributes to 50% mortality rate, and there is no effective treatment for the cardiomyopathy caused by DOXO [4]. The occurrence rate of the cardiomyopathy is related to the cumulative dose. For example, when the dose of DOXO is 500–550 mg/m^2^, the incidence is about 4%; however, when the dose exceeds 600 mg/m^2^, the incidence escalates to 36% [4]. 

DOXO is believed to cause cardiomyopathy via several ways, including the downregulation of genes for the contractile proteins, oxidative stress, and P_53_-mediated apoptosis [4]. Concerning the cardiac contractile dysfunction which occurs in DOXO-related cardiomyopathy, research interests have been focused on the interference of DOXO with intracellular Ca^2+^ homeostasis [5,6]. It is well known that the intracellular Ca^2+^ concentration is synergically regulated by several consecutive events: the entry of the extracellular Ca^2+^ via the voltage-gated Ca^2+^ channel (VGCC), which is activated following the generation and propagation of the action potential (AP); interaction of the Ca^2+^ ion with several intracellular molecules, eventually causing intracellular Ca^2+^ release via the ryanodine receptor Ca^2+^-releasing channel (RYR), which is located on the sarcoplasmic reticulum (SR); and the SR Ca^2+^-ATPase (SERCA) and Na^+^-Ca^2+^ exchanger (NCX) which are the major proteins that handle Ca^2+^ sequestering [7]. Changes in these Ca^2+^ regulatory proteins have been confirmed in rodents with DOXO injection [8,9]. The data relating to the intracellular Ca^2+^ level in cardiomyopathy caused by DOXO are discordant. Most of the current evidence tends to relate Ca^2+^ overload with the cardiomyopathy caused by DOXO [10,11,12,13]; however, Sag and Kohler et al. found a reduced intracellular Ca^2+^ level after DOXO incubation in isolated rat ventricular cardiomyocytes [14], and LIach et al. reported a decreased intracellular Ca^2+^ level with slowed rising velocity of the Ca^2+^ signal after 15 weeks of DOXO injection. This is concomitant with their observation of the reduced ejection fraction [15]. 

Tachycardias can lead to ventricular contractile dysfunction, and rapid pacing is widely used to produce animal models of cardiomyopathy [16] and atrial fibrillation [17]. Abnormalities of cardiac Ca^2+^ handling is one of the proposed mechanisms underlying the pacing-induced cardiac dysfunction. A downregulation of sarcoplasmic reticulum Ca^2+^ transport ATPase and myofibrillar Ca^2+^ ATPase has been documented in tachycardia-induced cardiomyopathy [18]. Although the abnormality in Ca^2+^ handling is a potentially unifying mechanism, controversy exists with regard to systolic dysfunction. Some groups suggest a decreased Ca^2+^ sensitivity [19], while others speculate abnormalities in excitation–contraction coupling that might be responsible for the systolic dysfunction [20]. 

In this study, we simultaneously optically mapped the dynamic signals of membrane potential and intracellular Ca^2+^ on an ex vivo Langendorff guinea pig heart, and the effects of DOXO and tachypacing were evaluated. We found that DOXO decoupled the temporal connection between the signals of AP and Ca^2+^. DOXO also decreased the rising and falling velocity of the Ca^2+^ signal, suggesting detrimental effects on machineries corresponding to both Ca^2+^ releasing and sequestering. Similar to the effect of DOXO, tachypacing also decoupled the AP-Ca^2+^ connection and slowed down the rising and falling velocity of the Ca^2+^ signal. In the rapid-paced group, however, the effects of DOXO were in contrast to that of the sinus group: 1 µM and 3 µM DOXO promoted the coupling between AP and Ca^2+^, and accelerated both the rising and falling velocity of the Ca^2+^ signal. The decreasing effect of DOXO on the intracellular Ca^2+^ signal might reveal, at least partially, the contractile dysfunction which occurs in DOXO-related cardiomyopathy, and the different effects of DOXO on the sinus and tachypaced heart suggested that DOXO might exert different effects on the heart with physiological and pathological status.

## 2. Methods

The guinea pig hearts were harvested from male adult animals (300~400 g) which were housed in the animal center of Tongji Medical College, Huazhong University of Science and Technique, and fed ad libitum (20~30 °C, 60% humility, 12:12 light/dark cycle). All experimental procedures with guinea pigs were approved by the Animal Welfare Committee of Huazhong University of Science and Technology, and experiments in this study were carried out in accordance with the rules of this committee.

Heparin (3125 IU/Kg) was administrated (intraperitoneal) 15 min before inhaling of Isoflurane. After being overdosed, the animal was scarified and their heart exposed. The heart was Langendorff-perfused with 37 °C KH solution (in mM: NaCl 119, KCl 4, CaCl_2_ 1.8, MgCl_2_ 1, NaH_2_PO_4_ 1.2, NaHCO_3_ 25, Glucose 10. pH = 7.4) for 15 min. The flow rate was set at 30 mL/min. Blebbistatin (6 mg/L), an excitation–contraction uncoupling agent, was loaded to arrest the heart, which ensures mechanical stabilization for consequential optical sampling. 

The dyes Rhod-2AM (1 mg/L) and RH273 (1 mg/L) were used for labelling Ca^2+^ and voltage signals, respectively, and Pluronic F127 (500 µg/L) was used as a dye solubilizer to facilitate their loading. These agents were circulating the heart for 15 min before sampling. 

A 530 ± 25 nm laser light source, LEDC-2001 (MappingLab, Oxford, UK), was applied for signal excitation. The emission light was detached via a dichroic mirror at 630 nm, and the Ca^2+^ and voltage signals were filtered at 590 nm and 700 nm before being sampled with two separate CMOS cameras (OMS-PCIE-2002, Oxford, UK, MappingLab). The sampling frequency was 0.8 kHz. The software package for recording was OMapScope4.0 (MappingLab, Oxford, UK). The Ca^2+^ and voltage signals were recorded simultaneously, so that their temporal properties could be evaluated. 

**Figure 1:**
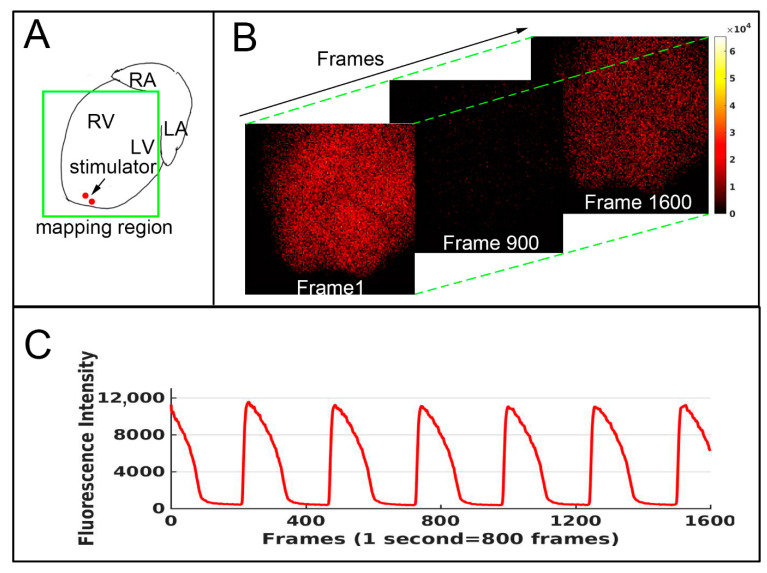
(**A**) schematic diagram showing the mapping region and the location of the pacing stimulator. (**B**) Consecutive mapping of the Ca^2+^ fluorescence signal. The sampling frequency was 800 frames/second. For clarity, only the 1st, 900th and 1600th frames are displayed. (**C**) Plot of the averaged fluorescence intensity of each frame in (**B**) (*Y* axis) versus the number of frames (*X* axis).

The sampling framing was 150 × 150 pixels. Each sampling episode lasted for one or two seconds, followed by a 5 min interval. The heart was subjected to two different excitation patterns: uninterfered sinus excitation and paced excitation. To pace the heart, a pair of stimulatory electrodes was placed on the apex, and a 5 Hz 2 mA current was applied. As illustrated in Figure 1A, signals from both left and right ventricles were mapped. 

Data were retrieved with custom-written Python code (Python 3.9) and analyzed with Matlab 2017a. As exampled in Figure 1B, the Ca^2+^ intensity signal was consecutively mapped onto 1600 frames; for clarity, only three of them (1, 900 and 1600) are displayed. The fluorescence intensity of each pixel can be extracted and plotted along the time axis (the frames). Thus, for each of the 150 × 150 segmented regions, the periodic oscillation of both Ca^2+^ and membrane potential can be monitored. As an example, Figure 1C shows the dynamic variation in the averaged Ca^2+^ signal of the mapping region. The peaks were detected; each of them marked one rhythmic activity. From each of these activities, the following information was retrieved: the maximum rising velocity (MRV), maximum falling velocity (MFV), and the 50% signal duration (CaD50) (the duration with intensity ⩾50% amplitude). Because voltage and Ca^2+^ signals were recorded simultaneously, the time difference of peak occurrence of Ca^2+^ and membrane potential (Ca-AP-Lag) could be calculated, which denotes the property of action potential–Ca^2+^ coupling. 

Blebbistatin and Rhod-2 AM were from Abcam (Cambridge, UK) and Rh-237 was from Univ (Shanghai, China). All other agents were from Sigma (Shanghai, China). 

The data were analyzed with the built-in statistical tool in GraphPad Prism 9.3 (GraphPad Software, CA, USA), and presented as mean ± SEM. An unpaired t-test, if not stated, was applied to compare the differences between groups, and the difference with *p* < 0.05 was regarded as significant. Figures were plotted with Prism or Matlab. 

## 3. Result

### 3.1. DOXO Delayed the Ca^2+^ Activation in Respect of the Occurrence of AP

It is well known that the rise in the intracellular Ca^2+^ level is a subsequent event following the generation of AP. Several events are involved in the AP-Ca^2+^ coupling: the entry of the extracellular Ca^2+^ via the voltage-gated Ca^2+^ channel and binding of the Ca^2+^ with Ryanodine receptors which induced Ca^2+^ release from the SR. The time lag between the Ca^2+^ and AP (Ca-AP-Lag, defined as the time difference between the peak time of AP and the peak time of Ca^2+^ signal) reflects the dynamics of the underlying AP-Ca^2+^ coupling events. 

**Figure 2:**
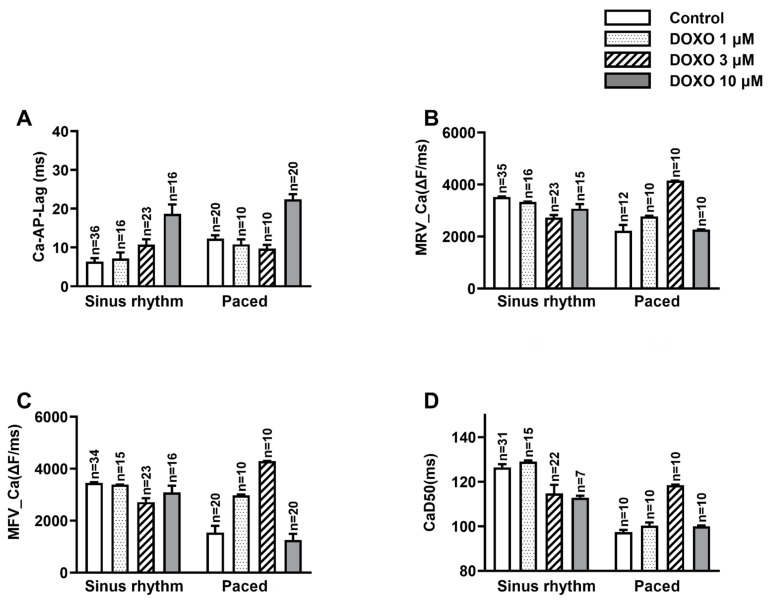
(**A**–**D**) the effect of DOXO on Ca-AP-Lag, MRV_Ca, MFV_Ca and CaD50 in sinus and tachypaced groups. (**A**) in the sinus group, 3 µM and 10 µM DOXO significantly prolonged the Ca-AP-Lag (*p* < 0.01); in the paced group, low concentration of DOXO (1 µM and 3 µM) had the opposite effect to that of 10 µM DOXO. (**B**) the MRV_Ca was decreased by DOXO (1 µM, 10 µM and 3 µM, *p* < 0.01) in the sinus group but increased in the tachypaced group by DOXO (1 µM and 3 µM, *p* < 0.05). (**C**) the MFV_Ca was decreased by DOXO (3 µM, *p* < 0.01 and 10 µM, *p* < 0.05) in the sinus group, but increased by DOXO (1 µM and 3 µM, *p* < 0.01) in the tachypaced group. (**D**) The CaD50 was decreased by DOXO (3 µM, *p* < 0.05 and 10 µM, *p* < 0.01) in the sinus group, but was increased by DOXO (10 µM, *p* < 0.01) in the tachypaced group. Ca-AP-Lag: the time difference between the peak of AP and the peak of the following Ca^2+^ signal; MRV_Ca and MFV_Ca: the maximum rising and following velocity of the Ca^2+^ signal; CaD50: the duration of the Ca^2+^ signal at 50% of the amplitude. ΔF: change in the fluorescence intensity.

We found that in hearts with sinus rhythm, DOXO prolonged the Ca-AP-Lag in a concentration-dependent manner. As shown in Figure 2A, the Ca-AP-Lag before the application of DOXO was 6.3 ± 0.9 ms, which was significantly increased to 10.8 ± 1.4, and 18.7 ± 2.4 ms after the treatment of DOXO 3 µM and 10 µM, respectively (*p* < 0.01, control vs. DOXO treatment). 

Compared to sinus rate excitation, pacing significantly increased the Ca-AP-Lag (Figure 2A). Although the ratio of the heart rate enhancement was not very high (from 272 ± 4 bpm (beats per minute) at sinus rate, to 300 bpm, with paced rate) regarding the high basal heart rate, fast pacing significantly increased the Ca-AP-Lag from 6.3 ± 0.9 to 12.4 ± 1.2 ms (*p* < 0.01, sinus control vs. paced control). In other words, fast pacing hampered the proper coupling between AP and Ca2+ signaling.

In the paced group, however, DOXO showed different effects compared with those in the sinus group. Although the *p* value did not reach the significant level, the low concentration of DOXO (1 uM and 3 µM) showed a trend of concentration-dependent decreasing effect on the Ca-AP-Lag (*p* = 0.06, control vs. DOXO 3 µM), opposite to its increasing effect on CA-AP-LAP in the sinus group. The higher concentration of DOXO (10 µM) in the tachypaced group, however, consistent with its effect in the sinus group, increased the Ca-AP-Lag (*p* < 0.01). In other words, a low concentration of DOXO tends to alleviate the pacing damaged Ca-AP coupling, while a high concentration of DOXO exacerbates the damage.

### 3.2. DOXO Hampered Both the Releasing and Sequestering Processes of Ca^2+^

The maximum rising velocity of the Ca^2+^ (MRV_Ca) signal reflects the process of Ca^2+^ release, and the maximum falling velocity of the Ca^2+^ signal (MFV-Ca) denotes the Ca^2+^-sequestering process. In the sinus group, DOXO treatment damaged both processes. For instance, 10 µM DOXO significantly reduced the MRV-Ca from 3520 ± 24 to 3072 ± 177 ΔF/ms (*p* < 0.01), and the MFV_Ca from 3460 ± 25 to 3094 ± 255 ΔF/ms (*p* < 0.05).

Tachypacing (300 bpm) is detrimental to both the releasing and the sequestering process of Ca^2+^. It reduced MRV-Ca from 3520 ± 24 (sinus rhythm) to 2225 ± 330 ΔF/ms (*p* < 0.01), and MFV_Ca from 3460 ± 25 (sinus rhythm) to 1541 ± 382 ΔF/ms (*p* < 0.01), respectively (Figure 2B,C). 

DOXO treatment had contrary effects on the pacing-interfered MRV_Ca and MFV_Ca. In the pacing group, 1 µM and 3 µM DOXO increased the MRV_Ca and MFV_Ca in a concentration-dependent manner, an action opposite to that observed in the sinus group. The higher concentration of DOXO (10 µM), however, pulled both the MRV_Ca and MFV_Ca down (Figure 2B,C). Thus, the low concentration of DOXO (1 and 3 µM) treatment accelerated the pacing slowed MRV_Ca and MFV_Ca; this might cause Ca^2+^ overload. The higher concentration (10 µM), however, fostered the slowing effect of pacing and might reduce the risk of Ca^2+^ overload. 

In the sinus group, DOXO treatment increased the duration of the Ca^2+^ signal (Figure 2D). The duration of the Ca^2+^ signal at 50% Ca^2+^ signal amplitude (CaD50) was significantly reduced by DOXO (3 and 10 µM) treatment. For example, the CaD50 before and after 10 µM DOXO was 126.5 ± 1.4 and 112.8 ± 0.9 ms (*p* < 0.01). In comparison with the sinus group, pacing (300 bpm) significantly shortened the CaD50 from 126.5 ± 1.4 to 97.3 ± 1.5 ms (*p* < 0.01). Within the paced group, however, DOXO 3 µM increased the CaD50 from 97.3 ± 1.5 to 118.5 ± 0.4 (*p* < 0.01), and DOXO 10 µM pulled the CaD50 down to 99.9 ± 0.5 ms. 

### 3.3. DOXO Increased the Heterogeneity of the CaD50

**Figure 3:**
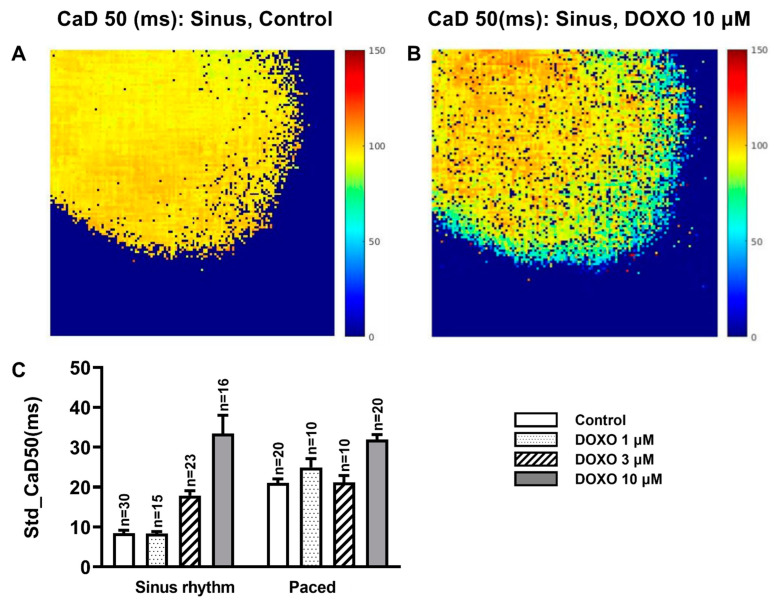
(**A**,**B**) An example mapping of the Ca^2+^ signal duration at 50% amplitude (CaD50) before (**A**) and after (**B**) the application of DOXO 10 µM in a heart with sinus rhythm. (**C**) The standard deviation (STD) of CaD50 in the sinus and paced groups. In the sinus group, DOXO 3 µM and 10 µM significantly increased the STD (*p* < 0.01), and in the paced group, DOXO 10 µM significantly increased the STD (*p* < 0.01). Compared to the sinus group, tachypacing (300 bpm) increased the STD (*p* < 0.01, sinus control vs. paced control).

As explained in the method (Figure 1), the parameters of the Ca^2+^ signals were retrieved for each of the mapped pixels (150 × 150); as a result, the heterogeneity of these parameters and the effects of tachypacing and DOXO treatment can be evaluated along the dimension of the spatial frame. The standard deviation (STD) was employed as a describer of the heterogeneity. As an example, Figure 3A shows the distribution pattern of the CaD50 (sinus rhythm, control) along the sampling frame, and Figure 3B displays the CaD50 after 10 µM DOXO treatment. It was clear that the variation in the signal was increased by DOXO. 

As shown in Figure 3C, the heterogeneity of CaD50 was increased by both DOXO treatment and tachypacing. The STD of CaD50 was significantly increased from 8.5 ± 0.7 to 33.5 ± 4.6 and 21.1 ± 1.4 ms by DOXO 10 µM treatment and tachypacing, respectively (*p* < 0.01, in both cases). In the tachypaced group, DOXO 10 µM also increased the heterogeneity of CaD50. 

DOXO and pacing did not affect the heterogeneity property of MRV and MRF (data not shown).

### 3.4. Correlation Analysis of the Ca^2+^ Properties

**Figure 4:**
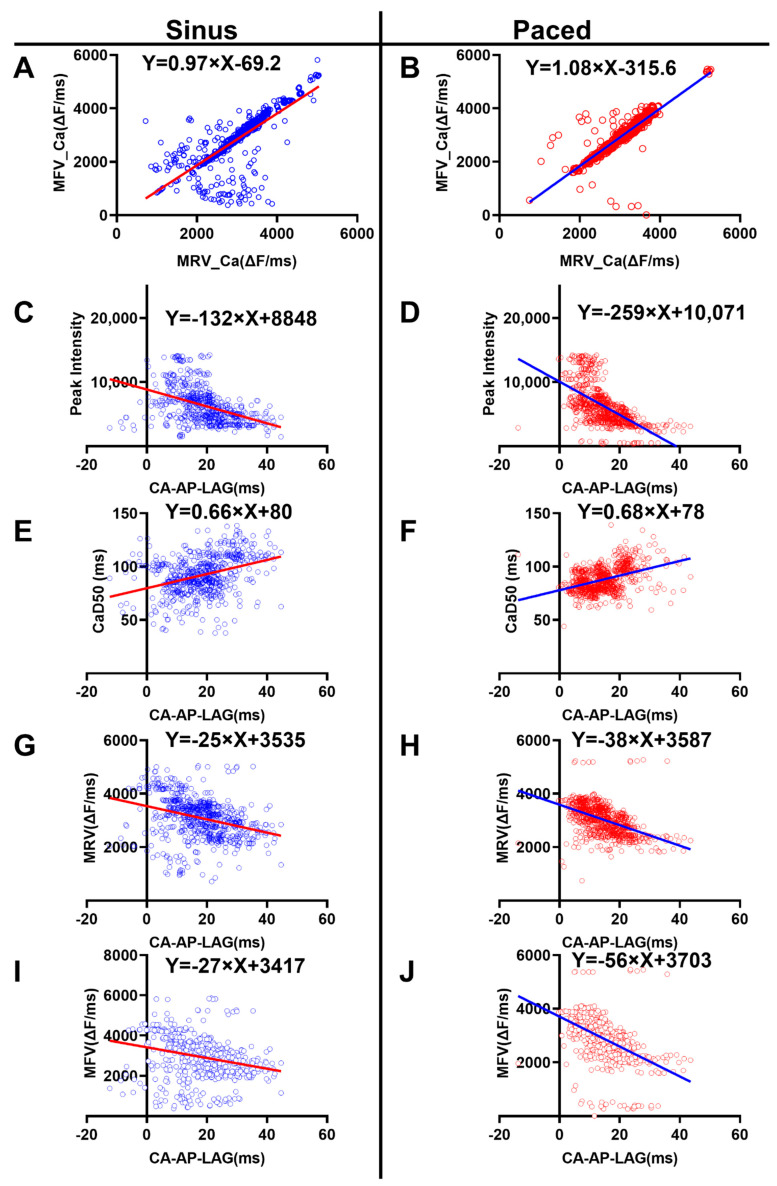
(**A**,**B**) The correlation of MRV_Ca and MFV_Ca in sinus group (**A**) and paced group. (**C**–**J**) the correlation of Ca-AP-Lag with the peak of Ca^2+^ signal ((**C**): sinus, (**D**): paced), CaD50 ((**E**): sinus, (**F**): paced), MRV ((**G**): sinus, (**H**): paced) and MFV ((**I**): sinus, (**J**): paced). All the correlations were performed with Pearson analysis and were significant (*p* < 0.01); and the equation Y = A × X ± B was employed to describe the simple linear regression (insets in each Figure).

In order to unveil the possible causes and/or effects of the change in Ca-AP-lag, a series of correlation analyses (Pearson) were performed between different Ca^2+^ properties. All the correlations in the results displayed in Figure 4 were significant (*p* < 0.05), and each were successfully fitted with a simple linear regression method: Y = A × X ± B, where X and Y represent the *x* and *y* axis data, and A and B are the slope factor and the Y-intercept, respectively.

1. The MRV_Ca was positively correlated with MFV_Ca

The correlation analysis revealed that the MRV and MFV of the Ca^2+^ signal were positively correlated with each other in both the sinus (Figure 4A) and the paced (Figure 4B) groups. Thus, myocytes with slow MRV Ca^2+^ signals also have slow MFV. In the heart with sinus rhythm, the correlation was successfully described by the equation: Y = 0.97×X −69.2, where X and Y represented the MRV and MFV, respectively. In fast-paced preparations, the linear equation was altered to Y = 1.08 × X − 316. This increase in the slope factor (from 0.97 to 1.08) suggested that, under the paced circumstance, a unit decrease in MRV corresponded to a more prominent decrease in MFV. This effect might contribute to, at least partially, the Ca^2+^ overload observed in the fast-paced heart model.

2. The Ca-AP-Lag was negatively correlated with the peak amplitude of Ca^2+^ signal

As illustrated in Figure 4C,D, the Ca-AP-Lag was negative correlated with the peak amplitude of Ca^2+^ in both groups of sinus rhythm (Figure 4C) and fast pacing (Figure 4D). Thus, a higher value of the Ca-AP-Lag corresponds to a smaller peak Ca^2+^ signal. In other words, myocytes with delayed Ca^2+^ activation had a smaller peak Ca^2+^ amplitude; thus, DOXO, that slowed down the Ca^2+^ activation, might also reduce the amount of Ca^2+^ entry/release during the activation phase. 

The correlation was stronger in the pacing group than that in the sinus group, as the slope factor was increased from −132 (the sinus group) to −259 (the tachypaced group). Therefore, similar change in the Ca-AP-LAG corresponds to a more prominent change in the peak Ca^2+^ signal.

3. The Ca-AP-Lag was positively correlated with duration of the Ca^2+^ signal

The Ca-AP-Lag was positively correlated with CaD50 in both the sinus (Figure 4E) and paced (Figure 4F) groups, with similar slope factors (0.66 and 0.68 for the sinus and paced groups, respectively). Thus, myocytes with a delayed rise in the Ca^2+^ signal also displayed an increased Ca^2+^ activation window. This might contribute to the reported DOXO-related Ca^2+^ overloading which underlines the toxic effect of DOXO treatment.

4. The Ca-AP-Lag was negatively correlated with the Ca^2+^ MRV and MFV

The Ca-AP-Lag was negatively correlated with the Ca^2+^ MRV and MFV in both sinus and fast-paced groups (Figure 4G–J). The correlations for MRV were described with the equation of Y = −25 × X ± 3535 in the sinus group and Y = −38 × X ± 3587 in the fast-paced group; the correlations for MFV were described with the equation of Y = −27 × X ± 3417 in the sinus group and Y = −56 × X ± 3703 in the fast-paced group. These results indicated that a delayed activation of Ca^2+^ coexisted with slowed releasing and sequestering dynamics of Ca^2+^, and this effect was more prominent in the tachypaced group. 

## 4. Discussion

The current research evaluated the effect of DOXO, tachypacing and DOXO–tachypacing interactions on the dynamics of intracellular Ca^2+^ signals. The membrane potential and Ca^2+^ signal were optically mapped and performed ex vivo on Langendorff guinea pig hearts. The main finding was that DOXO treatment favored a reduction in the intracellular Ca^2+^ level; this was suited well to explaining the mechanism of the contractile dysfunction which occurs in DOXO-related cardiomyopathy.

Our results suggested a retarding effect of DOXO on one or several of the essential targets that contribute to the rising (for instance, VGCC and RYR) and falling (for instance, SERCA and NCX) of the intracellular Ca^2+^ signal (Figure 2A). Together with the observations that DOXO shortened the Ca^2+^ duration, CaD50 and the negative correlation between Ca-AP-Lag and the peak of the Ca^2+^ signal were observed. Our results support a hypothesis that DOXO treatment reduced the intracellular Ca^2+^ level; this might be responsible for the systolic contractile dysfunction presented in DOXO-related cardiomyopathy [4]. This was consistent with the work of Sag, C.M. et al. [14] and LIach, A. et al. [15] whose work indicated a reduced intracellular Ca^2+^ transience after the administration of DOXO. Moreover, the observations of the reduced RYR expression in the presence of DOXO [9,21] might contribute to the slowed MRV observed in the current research. Although, the current mainstream supports an opinion that DOXO might provoke Ca^2+^ overload, which causes lesions presented in cardiomyopathy [10,11,12,22,23]. This opinion, however, was disputable in terms of elucidating the contractile cardiac dysfunction. 

Several possible factors might contribute to this discrepancy between the current research and the available data. The first consideration is the recording method. In the current experiment, the Ca^2+^ signal was directly measured with optical procedure at the whole-heart level. Many previous works were performed on the isolated cardiomyocytes [22,23]; their properties might be quite different from the in vivo or ex vivo hearts. Moreover, the species differences should also be considered. For example, contrary to our observation that DOXO shortened the CaD50, the work of Chao et al., in isolated zebrafish cardiomyocytes, found that DOXO prolonged the Ca^2+^ transient and increased the sarcomere contraction [13]. More importantly, for some experiments, the level of the Ca^2+^ signal was determined at the tissue level, and the static total Ca^2+^ amount was measured due to a technique limitation at the time of the experiment [10,12]. As a result, the measured Ca^2+^ level might not precisely reflect the Ca^2+^ level in the cytosol rather than the total amount in all compartments of the cell, and the essential dynamic characteristics of the cytosol Ca^2+^ were overlooked. Additionally, for some research, the conclusion of an increased intracellular Ca^2+^ might be based on assumptions from some ‘indirect’ observations; for instance, in isolated rat cardiomyocytes, whole-cell patch clamp recording revealed that DOXO increased the amplitude of the L-type Ca^2+^ current [23], and some research confirmed that DOXO reduced the expression of SERCA and RYR at the mRNA or protein level [8,9]. 

Importantly, the work of Ondrias, K. et al. [24] confirmed that DOXO had biphasic effects on the cardiac SR Ca^2+^-releasing channel incorporated in an artificial lipid bilayer. The initial exposure of DOXO increased the opening probability of the channel; after a mean of 8 min DOXO, however, it irreversibly inhibited the channel. Because the DOXO-related cardiomyopathy occurred after chronic treatment, the general effects of DOXO would more likely inhibit the SR Ca^2+^ channel. This was consistent with our observations. Interestingly, the pretreatment of dithiothreitol (DTT) prevented the inhibiting effect of DOXO on the SR Ca^2+^ channel [24].Because of the high efficiency of DTT in restoring the function of sulfhydryl groups damaged by reactive oxygen species (ROS),wehypothesized that the direct effect of chronic DOXO treatment was to inhibit the SR Ca^2+^-releasing channel, and, thus, reduce the intracellular Ca^2+^; calcium overloading might be an indirect effect of DOXO. For instance, DOXO might promote the release of ROS [14] which altered the response of calcium-handling proteins to DOXO [25].

Although the current work did not support the mechanism of Ca^2+^-overload-related DOXO cardiotoxicity, the increased heterogeneity of the Ca^2+^ signal might play a role. The heterogeneities of the heart electrical properties were greatly concerned, because the abnormal increase in many of them was proarrhythmic [26,27,28,29]. For instance, the spatial variability of the repolarization of the ventricular myocardium has been linked to the arrhythmogenesis through reentry and increased dispersion of refractories, and alternans in intracellular Ca^2+^ handling underlined the alternans in AP morphology [30]. Therefore, our finding that DOXO increased the heterogeneity of CaD50 might contribute to the arrhythmogenesis in DOXO treatment [31,32]. Similarly, Kharin, S. et al. [33], with a method of 64-electrode array ventricular epicardial mapping, confirmed an increased heterogeneity of ventricular repolarization after chronic DOXO treatment.

It was important to point out that the role of Ca^2+^ overload in DOXO-related cardiomyopathy cannot be ruled out based on the current research. Firstly, the slowing effect of DOXO in the falling velocity of the Ca^2+^ (MFV_Ca) (Figure 2C) might contribute to the possible development of Ca^2+^ accumulation. This was consistent with the observation that DOXO decreased the expression of SERCA and increased the level of PLB [8,9,34]. Secondly, multivariables, especially the dosage and the basal condition of the heart, are involved in the development of the cardiomyopathy. A different dosage of DOXO might have unanimous effects on the Ca^2+^ homeostasis in hearts with different basal status [8,35]. For example, in the tachypaced group, a low concentration of DOXO (1 uM and 3 µM) generally displayed the opposite effect in terms of that in the sinus group (Figure 2). Their tendency of increasing MRV and CaD50 might lead to Ca^2+^ overload in the tachypaced heart.

Limitations and future works: Although the current work stated a general reducing effect of DOXO on the intracellular Ca^2+^ level, further investigations, however, need to be carried out to clarify the underlying mechanisms. As a series of cascade molecules are involved in the fine-tuning of the intracellular Ca^2+^, it was valuable to test the effects of these molecules on the DOXO-meddled hearts. For instances, DOXO is known to affect Ca^2+^ homeostasis by modulation of SERCA and NCX, and the NCX inhibitor ameliorates DOXO toxicity in cardiomyocytes [25]. Thus, investigation of the effects of SERCA and NCX inhibitors would be helpful to unveil the mechanism of the DOXO toxicity. Moreover, as DOXO also affected gene expression of the intracellular Ca^2+^ handling proteins, such as RYRs and SERCA [8,9], it was helpful to evaluate how DOXO at the RNA and/or DNA level affects the intracellular Ca^2+^ level. 

The results of this study demonstrate the effect of DOXO in reducing the intracellular Ca^2+^ level, providing experimental evidence for elucidating the long-existing controversy in terms of the contractile dysfunction in DOXO-caused cardiomyopathy. Furthermore, DOXO in the tachypaced hearts had the opposite effect to that in hearts with sinus rhythm. This suggested the importance of Ca^2+^-overload-related cardiotoxicity in a heart undergoing pathological states. Although more affirmative research still needs to be performed, the current research might shed some light on the clinical strategies for treating and/or preventing DOXO-induced cardiomyopathy.

## 5. Conclusions

In heart with sinus rhythm, DOXO led to Ca^2+^ underload; this might contribute to the contractile dysfunction in DOXO-related cardiomyopathy. In the tachypaced hearts, however, DOXO might increase the intracellular Ca^2+^ level and cause Ca^2+^-overload-related toxicity.

## Data Availability

Data is contained within the article.

## References

[1] Cho H., Zhao X.X., Lee S., Woo J.S., Song M.Y., Cheng X.W., Lee K.H., Kim W. (2022). The sGC-cGMP Signaling Pathway as a Potential Therapeutic Target in Doxorubicin-Induced Heart Failure: A Narrative Review. Am. J. Cardiovasc. Drugs.

[2] Gao H., Xian G., Zhong G., Huang B., Liang S., Zeng Q., Liu Y. (2022). Alleviation of doxorubicin-induced cardiomyocyte death through miR-147-y-mediated mitophagy. Biochem. Biophys. Res. Commun..

[3] Jang H.M., Lee J.Y., An H.S., Ahn Y.J., Jeong E.A., Shin H.J., Kim K.E., Lee J., Koh J.S., Roh G.S. (2022). LCN2 deficiency ameliorates doxorubicin-induced cardiomyopathy in mice. Biochem. Biophys. Res. Commun..

[4] Chatterjee K., Zhang J., Honbo N., Karliner J.S. (2010). Doxorubicin cardiomyopathy. Cardiology.

[5] Agustini F.D., Arozal W., Louisa M., Siswanto S., Soetikno V., Nafrialdi N., Suyatna F. (2016). Cardioprotection mechanism of mangiferin on doxorubicin-induced rats: Focus on intracellular calcium regulation. Pharm. Biol..

[6] Shinlapawittayatorn K., Chattipakorn S.C., Chattipakorn N. (2022). The effects of doxorubicin on cardiac calcium homeostasis and contractile function. J. Cardiol..

[7] Bupha-Intr T., Wattanapermpool J. (2006). Regulatory role of ovarian sex hormones in calcium uptake activity of cardiac sarcoplasmic reticulum. Am. J. Physiol. Heart Circ. Physiol..

[8] Pecoraro M., Rodríguez-Sinovas A., Marzocco S., Ciccarelli M., Iaccarino G., Pinto A., Popolo A. (2017). Cardiotoxic Effects of Short-Term Doxorubicin Administration: Involvement of Connexin 43 in Calcium Impairment. Int. J. Mol. Sci..

[9] Olson R.D., Gambliel H.A., Vestal R.E., Shadle S.E., Charlier H.A., Cusack B.J. (2005). Doxorubicin cardiac dysfunction: Effects on calcium regulatory proteins, sarcoplasmic reticulum, and triiodothyronine. Cardiovasc. Toxicol..

[10] Olson H.M., Young D.M., Prieur D.J., LeRoy A.F., Reagan R.L. (1974). Electrolyte and morphologic alterations of myocardium in adriamycin-treated rabbits. Am. J. Pathol..

[11] Jaenke R.S. (1976). Delayed and progressive myocardial lesions after adriamycin administration in the rabbit. Cancer Res..

[12] Buhner R., Biedert S., Miura D. (1980). [Adriamycin-cardiomyopathy induced by an increment of (Ca^2+^)] (author’s translation). Klin. Wochenschr..

[13] Chao Y.K., Liau I. (2021). One-dimensional scanning multiphoton imaging reveals prolonged calcium transient and sarcomere contraction in a zebrafish model of doxorubicin cardiotoxicity. Biomed. Opt. Express.

[14] Sag C.M., Köhler A.C., Anderson M.E., Backs J., Maier L.S. (2011). CaMKII-dependent SR Ca leak contributes to doxorubicin-induced impaired Ca handling in isolated cardiac myocytes. J. Mol. Cell. Cardiol..

[15] Llach A., Mazevet M., Mateo P., Villejouvert O., Ridoux A., Rucker-Martin C., Ribeiro M., Fischmeister R., Crozatier B., Benitah J.-P. (2019). Progression of excitation-contraction coupling defects in doxorubicin cardiotoxicity. J. Mol. Cell. Cardiol..

[16] Shinbane J.S., A Wood M., Jensen D., A Ellenbogen K., Fitzpatrick A.P., Scheinman M.M. (1997). Tachycardia-induced cardiomyopathy: A review of animal models and clinical studies. J. Am. Coll. Cardiol..

[17] Schüttler D., Bapat A., Kääb S., Lee K., Tomsits P., Clauss S., Hucker W.J. (2020). Animal Models of Atrial Fibrillation. Circ. Res..

[18] O’Brien P.J., Ianuzzo C.D., Moe G.W., Stopps T.P., Armstrong P. (1990). Rapid ventricular pacing of dogs to heart failure: Biochemical and physiological studies. Can. J. Physiol. Pharmacol..

[19] Perreault C.L., Shannon R.P., Komamura K., Vatner S.F., Morgan J.P. (1992). Abnormalities in intracellular calcium regulation and contractile function in myocardium from dogs with pacing-induced heart failure. J. Clin. Investig..

[20] Wolff M.R., Whitesell L.F., Moss R.L. (1995). Calcium sensitivity of isometric tension is increased in canine experimental heart failure. Circ. Res..

[21] Gambliel H.A., Burke B.E., Cusack B.J., Walsh G.M., Zhang Y.L., Mushlin P.S., Olson R.D. (2002). Doxorubicin and C-13 deoxydoxorubicin effects on ryanodine receptor gene expression. Biochem. Biophys. Res. Commun..

[22] Miwa N., Kanaide H., Meno H., Nakamura M. (1986). Adriamycin and altered membrane functions in rat hearts. Br. J. Exp. Pathol..

[23] Keung E.C., Toll L., Ellis M., A Jensen R. (1991). L-type cardiac calcium channels in doxorubicin cardiomyopathy in rats morphological, biochemical, and functional correlations. J. Clin. Investig..

[24] Ondrias K., Borgatta L., Kim D.H., E Ehrlich B. (1990). Biphasic effects of doxorubicin on the calcium release channel from sarcoplasmic reticulum of cardiac muscle. Circ. Res..

[25] Tocchetti C.G., Carpi A., Coppola C., Quintavalle C., Rea D., Campesan M., Arcari A., Piscopo G., Cipresso C., Monti M.G. (2014). Ranolazine protects from doxorubicin-induced oxidative stress and cardiac dysfunction. Eur. J. Heart Fail..

[26] Aslanidi O.V., Sleiman R.N., Boyett M.R., Hancox J.C., Zhang H. (2010). Ionic mechanisms for electrical heterogeneity between rabbit Purkinje fiber and ventricular cells. Biophys. J..

[27] Sekar R.B., Kizana E., Cho H.C., Molitoris J.M., Hesketh G.G., Eaton B.P., Marban E., Tung L. (2009). IK1 heterogeneity affects genesis and stability of spiral waves in cardiac myocyte monolayers. Circ. Res..

[28] Yeh H.-I., Lai Y.-J., Lee S.-H., Lee Y.-N., Ko Y.-S., Chen S.-A., Severs N.J., Tsai C.-H. (2001). Heterogeneity of myocardial sleeve morphology and gap junctions in canine superior vena cava. Circulation.

[29] Antzelevitch C. (2000). Electrical heterogeneity, cardiac arrhythmias, and the sodium channel. Circ. Res..

[30] Nemec J. (2016). Nonalternans repolarization variability and arrhythmia—The calcium connection. J. Electrocardiol..

[31] Hazari M.S., Haykal-Coates N., Winsett D.W., Costa D.L., Farraj A.K. (2009). Continuous electrocardiogram reveals differences in the short-term cardiotoxic response of Wistar-Kyoto and spontaneously hypertensive rats to doxorubicin. Toxicol. Sci..

[32] Rudzinski T., Ciesielczyk M., Religa W., Bednarkiewicz Z., Krzeminska-Pakula M. (2007). Doxorubicin-induced ventricular arrhythmia treated by implantation of an automatic cardioverter-defibrillator. Europace.

[33] Kharin S., Krandycheva V., Tsvetkova A., Strelkova M., Shmakov D. (2013). Remodeling of ventricular repolarization in a chronic doxorubicin cardiotoxicity rat model. Fundam. Clin. Pharmacol..

[34] Pecoraro M., Ciccarelli M., Fiordelisi A., Iaccarino G., Pinto A., Popolo A. (2018). Diazoxide Improves Mitochondrial Connexin 43 Expression in a Mouse Model of Doxorubicin-Induced Cardiotoxicity. Int. J. Mol. Sci..

[35] Hanna A.D., Lam A., Tham S., Dulhunty A.F., Beard N.A. (2014). Adverse effects of doxorubicin and its metabolic product on cardiac RyR2 and SERCA2A. Mol. Pharmacol..

